# Carboxypeptidase A3—A Key Component of the Protease Phenotype of Mast Cells

**DOI:** 10.3390/cells11030570

**Published:** 2022-02-06

**Authors:** Dmitri Atiakshin, Andrey Kostin, Ivan Trotsenko, Vera Samoilova, Igor Buchwalow, Markus Tiemann

**Affiliations:** 1 Research and Educational Resource Center for Immunophenotyping, Digital Spatial Profiling and Ultrastructural Analysis Innovative Technologies, Peoples’ Friendship University of Russia, Miklukho-Maklaya Str. 6, 117198 Moscow, Russia; atyakshin-da@rudn.ru (D.A.); kostin-aa@rudn.ru (A.K.); trotsenko-id@rudn.ru (I.T.); 2 Research Institute of Experimental Biology and Medicine, Burdenko Voronezh State Medical University, Studencheskaya Str. 10, 394036 Voronezh, Russia; 3 Institute for Hematopathology, Fangdieckstr. 75a, 22547 Hamburg, Germany; verasamoilova@hotmail.com (V.S.); mtiemann@hp-hamburg.de (M.T.)

**Keywords:** mast cells, carboxypeptidase A3, secretome, granules, secretory pathways, specific tissue microenvironment

## Abstract

Carboxypeptidase A3 (CPA3) is a specific mast cell (MC) protease with variable expression. This protease is one of the preformed components of the secretome. During maturation of granules, CPA3 becomes an active enzyme with a characteristic localization determining the features of the cytological and ultrastructural phenotype of MC. CPA3 takes part in the regulation of a specific tissue microenvironment, affecting the implementation of innate immunity, the mechanisms of angiogenesis, the processes of remodeling of the extracellular matrix, etc. Characterization of CPA3 expression in MC can be used to refine the MC classification, help in a prognosis, and increase the effectiveness of targeted therapy.

## 1. Introduction

Mast cells (MC) as a multifunctional component of a specific tissue microenvironment, are in the focus of numerous studies in various biomedical areas. Identified by Paul Ehrlich more than 140 years ago, MCs have firmly taken the position of special cells of connective tissue, with a wide range of sensory and regulatory properties to cells and non-cellular structures of the extracellular matrix, realization of innate and acquired immunity. Differentiating from CD34+ cells of the red bone marrow, MCs enter the bloodstream and selectively colonize various tissues and organs [1,2,3,4].

The classification of human MC is based on the content of specific proteases-chymase and tryptase [5,6]. At the same time, along with them, carboxypeptidase A3 (CPA3) is no less abundant component of the mast cell secretome [7,8,9,10,11]. CPA3 is involved in the pathogenesis of cancer, inflammatory diseases of the gastrointestinal tract, respiratory and cardiovascular systems, disorders of the musculoskeletal system, as well as in immunogenesis. In particular, CPA3 can also be considered as a diagnostic marker and pharmacological target in the treatment of a number of pathological conditions [8,12,13,14,15,16,17,18,19,20,21,22,23,24,25,26,27,28,29,30]. In addition, CPA3 in serum appeared as a good biomarker for identifying severe COVID-19 patients [31].

Fundamental aspects of mast cell CPA3 processing are of great practical importance as a diagnostic object, pharmacological target, and a criterion for the effectiveness of therapy. The key work in the field of MC biology to systematize the morpho-functional features of MCA3 MC was carried out in 2009 [32] and by now the need has ripened to supplement the available information with new results.

## 2. Molecular Genetic Aspects

Information about human mast cell proteases accounts for about 5% of the genome, which allows the level of the total pool of the corresponding mRNA to be in comparable values with the results of the expression of housekeeping genes. Due to the high level of expression of proteases, they can account for one third of the total amount of proteins in the cytoplasm of MC [6,33,34,35]. CPA3 is one of the specific proteases of MC, and unlike tryptase or chymase, it is a zinc-containing metalloproteinase with exopeptidase activity [11]. The human CPA3 gene consists of 11 exons and is located on chromosome 3 (3q24), forming, together with the pancreatic carboxypeptidase B1 gene (CPB1), a separate locus “CPB1-CPA3”. This locus is located on the border with the region of the type II angiotensin receptor gene on the one hand and, on the other hand, with the GYG1 and HLTF genes. The GYG1 gene encodes the glycogenin-1 enzyme involved in glycogen synthesis, and the HLTF gene encodes a helicase-like transcription factor [11]. In MC granules, CPA3 can range from 0.5 to 16 mg/10^6^, depending on the organ [36]. At the same time, human CPA3 has homologous features of rodent CPA3 in terms of molecular organization and amino acid residues involved in the binding of zinc to the substrate [36,37]. To date, expression of the CPA3 gene has been found only in MC, with the possible exception of basophilic leukocytes in patients with an allergic history [7,37,38,39,40,41]. In rats, up to 0.5% of the total mRNA pool in the abdominal MC is CPA3 mRNA, exceeding the expression level of actin gene transcription [42].

The expression of the CPA3 gene can be characterized by high organ-specific variability. For example, in rodents, CPA3 expression was detected in a subpopulation of connective tissue in MCs located in the serous cavity, skin and submucosa of the digestive tract, and was not detected in MCs of the intestinal mucosa and respiratory tract [40,42,43,44,45]. In humans, the expression of CPA3 was detected in MCs in connective tissue, which, as a rule, have simultaneous biogenesis of tryptase and chymase [7,46,47]. At the same time, in our studies to identify specific MC proteases, we found that CPA3 can also be expressed in tryptase-positive human MCs with no chymase, including in the mucous membrane of the stomach and small intestine [48]. Similar data were obtained in a recent study showing different levels of CPA3 in MCs of the skin, lungs and intestines [10]. MCs with the expression of both specific proteases contained CPA3 in a greater amount than the MC phenotype “tryptase + chymase”. The presence of CPA3 in tryptase-positive MCs significantly complements the data on the functional significance of co-localization of CPA3 with chymase [7,10]. Moreover, in some pathological conditions, chymase-negative mast cells with CPA3 expression have been found [27,49,50,51].

Quantitative indicators of the intracellular content of CPA3 can be used as a marker of MC differentiation, since the content of the protease increases during maturation [43,52,53]. A certain role of GATA transcription factors in the regulation of CPA3 gene expression is known [54,55]. On the contrary, microphthalmia-associated transcription factor (MITF), which plays an important role in the regulation of chymase and tryptase genes in MC, does not affect transcription of the CPA3 gene [56]. It has also been shown that the expression of the CPA3 gene increases significantly under the influence of glucocorticoids [57,58]. In the lung MCs, a close relationship between the expression of the CPA3 gene and the mRNA content of tryptase, not chymase, was shown [10]. It was suggested that the expression of the CPA3 gene is influenced by stimuli associated with innate rather than adaptive immunity [7,32].

Biogenesis of CPA3 in MCs is realized in the course of successive intracellular stages of synthesis and ends with its gradual folding in granules in a complex with other components of the secretome. At the final stages of biogenesis, CPA3 becomes a form with biological activity. On the putative structure of the CPA molecule, by analogy with porcine pancreatic carboxypeptidase B, a globular three-layer α/β protein with a central eight-stranded β sheet covered on each face by a layer of α helices was predicted, in which the active site is located at the C-terminal end of the parallel β sheet structure [7,32]. At the same time, the predominance of positively charged amino acid residues (Arg, Lys, and His-charged residues) was assumed in the CPA3 molecule, which is important from the point of view of interaction with polyanions of MC secretory granules during intragranular folding, in particular, with heparin. Secretory granules as a source of CPA3 are unique intracellular structures with highly specific regulation of the activity of excretion of protease and other secretome components outside the maternal granule into the cytosol and extracellular matrix [7,32,59,60,61,62,63,64].

## 3. Biogenesis of CPA3-Containing Granules

The initial stages of the formation of MC granules occur in the endoplasmic reticulum in the process of translation and transport of proteins, including CPA3 and other components of the secretome. Maturation of specific MC proteases is accompanied by sequential movement from the cis-section to the trans-section of the Golgi complex, from which clathrin-coated transport vesicles are detached [65,66,67]. These “progranules” have similar small sizes and undergo fusion with each other, as well as with early endosomes, gradually increasing in volume [61]. The molecular mechanisms of these processes are still unclear, but a special role belongs to secretogranin III [62]. Further maturation of progranules is accompanied by a gradual accumulation of proteases and other components of the secretome in their matrix [52]. An important event in the maturation process is the acidification of the intragranular medium to pH = 5.5. According to one model, sorting of the components of the secretome of progranules can occur with the help of sequential selective inclusion of new products in the Golgi complex, while the other model describes the possibility of removing the unclaimed part of the mediators from the previously synthesized matrix. An example of the first option is the operation of a system with the participation of mannose-6-phosphate, which promotes the selective addition of a number of proteins, including lysosomal hydrolases, to the secretion using the glycosylation process [66,68]. The mechanisms of the inclusion of CPA3 into the intragranular matrix are still not clear enough, although serglycine may play a certain role in them [32,62,69,70]. This pattern is typical for processes of biogenesis of tryptase and chymase [48,71,72,73].

During the maturation of pro-CPA3, cysteine proteases, including lysosomal cathepsins, can participate in this process [74]. In this case, the cathepsins E, C, S present in the secretory granules are of particular importance [75,76]. The possibility of the final completion of post-translational changes in CPA3 directly in granules was considered in the work of Rath-Wolfson [77].

Pro-CPA3 processing depends on heparin associated with serglycine [78]. At the same time, an interesting fact is the possibility of intragranular processing of CPA3 to an active state, the degree of processing of pro-CPA3 into an active protease may depend on both the age of the cells and the whole organism [77,79]. Based on the data of electron microscopic and molecular biological studies, Dvorak A.M. with colleagues suggested the possible localization of RNA directly in the granules of MC [59,60,80,81,82]. In this case, the role of secretory granules significantly expands the understanding of both the intracellular mechanisms of secretome biogenesis and the extracellular pathways of CPA3 biogenesis with free localization of granules in the extracellular matrix.

Like chymase and tryptase in MCs, CPA3 is stored in secretory granules in active form. This has been shown for mature MCs, while in less differentiated MCs of the bone marrow pro-CPA3 with a preserved activation peptide, which can dominate the active form of the enzyme, was detected [78,79]. Low enzymatic activity during intragranular storage is provided by the acidic medium of the granules (pH 5.5), while the optimal pH for CPA3 activity is in the pH range from 7 to 9.

**Figure 1:**
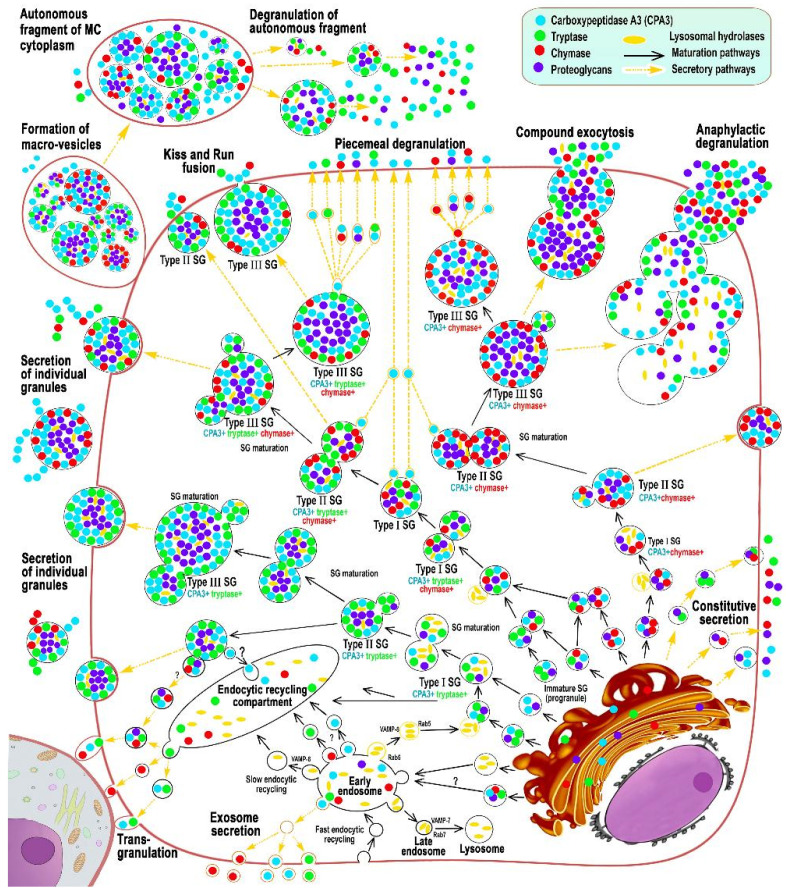
Main stages of biogenesis and secretory pathways of mast cell carboxypeptidase A3 (adapted from Atiakshin [84]). The diagram shows the main stages of post-translational modification of CPA3 with an emphasis on cytotopography, intragranular localization, and secretory mechanisms. CPA3 biosynthesis begins in the granular endoplasmic reticulum mast cells (MC) and continues in the Golgi apparatus (GA), where the progranule is formed. Secretory granules (SG) can be classified into 3 types. Type I SGs are formed after fusion of lysosomes with progranules, budding from GA, and have the smallest size and initial CPA3 content. The size of type I granules can increase due to homotypic fusion with the formation of type II granules with a size of 0.2–0.4 μm. Type II secretory granules already possess a certain phenotype of specific proteases. As a result of the completion of maturation, type III SGs are formed with a size of 0.5 μm and more, which are characterized by the largest volume of secretome and peripheral localization of CPA3 in the form of a ring. The main mechanisms of CPA3 secretion from MC into the extracellular matrix are piecemeal degranulation, transgranulation, «kiss-and-run», exosome formation, exocytosis of individual secretory granules, as well as the formation of macrovesicles that retain the ability for autonomous secretion of proteases in a specific tissue microenvironment.

Thus, in the process of biogenesis of secretory granules in the trans-section of the Golgi complex, post-translational modifications of the protein component of the secretory product are partially completed, due to the range of necessary regulatory effects in relation to the structures of a specific tissue microenvironment. The functioning of the Golgi complex as a protein sorter during the formation of lysosomes, the inclusion and modification of intragranular proteoglycans, the provision of active sulfation of both carbohydrate and protein components implements constitutive and inducible secretory mechanisms [66,67,83]. After budding from the Golgi complex, the secretory progranules are still small; however, as a result of repeated fusion with each other, larger immature granules are formed, with the peculiarities of the composition of the central and peripheral regions [62]. Since MC granules are rich in a wide range of lysosomal hydrolases, they are called secretory lysosomes. Ripening of granules is accompanied by the formation of a denser central region and gradual filling of granules with proteases, bioactive amines, cytokines, etc. In this case, proteoglycans, which are essential for the maturation of granules, are essential for selective sorting with selective accumulation of secretome components, including CPA3 secretome components, including CPA3 [52,62,83], see Figure 1.

For induced secretion, the formation of granules is accompanied by the formation of larger supramolecular aggregates in the Golgi complex, the gradual maturation of which is accompanied by the loss of auxiliary enzymes, a gradual decrease in the intragranular pH level, and the selective accumulation of secretome components, including specific proteases and proteoglycans [52,85,86,87,88]. In addition, the fusion of the progranule with the late endosome leads to the division of the contents of the modified lysosome into a zone with a high density of packed structures surrounded by luminal vesicles [89] (Figure 1). The further process of granule maturation is associated with selective aggregation and accumulation of regulatory proteins in the central region, which is represented by proteoglycans from the moment of entry from Golgi complex. This “nucleus” can be considered an important coordinating link in the subsequent stages of granule maturation, both from the histotopographic and functional points of view. In this case, a complex of serglycine with heparin or chondroitin sulfate accumulates in the center of the granule [90]. Morphological evidence of this fact is the smaller size of metachromatic MC granules after histochemical staining, for example, with toluidine blue, in comparison with the immunomorphological detection of specific mast cell proteases [91]. It should be noted that serglycine is a necessary proteoglycan of the granule for the subsequent folding of specific MC proteases, as well as histamine and serotonin. As an explanation for this phenomenon, one can consider the existence of electrostatic interactions between sulfated and, as a result, negatively charged glycosaminoglycan molecules in a complex with serglycine, and positively charged sites of specific proteases [62].

**Figure 2:**
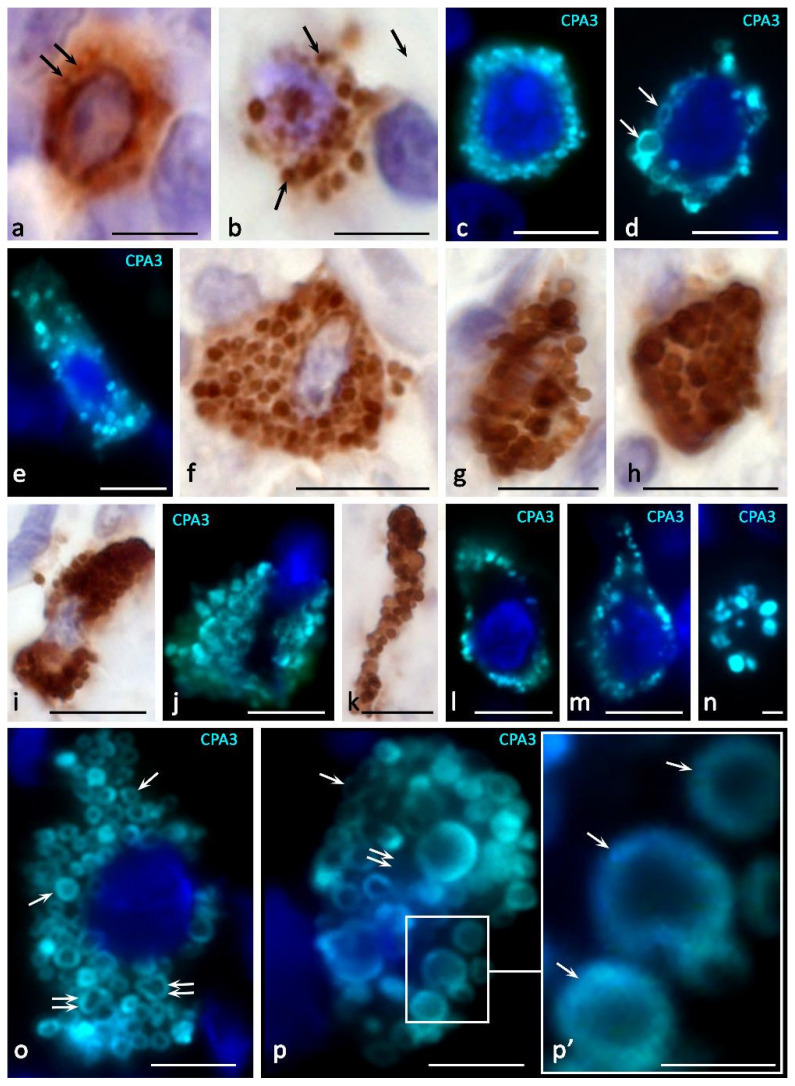
Cytotopography and morphological features of CPA3 MC biogenesis. Primary antibodies used: Rabbit polyclonal to CPA3 antibody (ab251696) (AbCam, Cambridge, UK). Secondary antibodies used: (**a**,**b**,**f**–**i**,**k**) AmpliStain anti-Rabbit 1-Step HRP (#AS-R1-HRP), SDT GmbH, Baesweiler, Germany, label-HRP. (**c**–**e**,**j**,**l**–**p**) Goat anti-rabbit IgG Ab (#A-11034) Invitrogen Darmstadt, Germany, label-Cy3. (**a**) Tonsilla. Initial stages of intragranular packing of CPA3 in MC (indicated by an arrow). (**b**) Stomach. Formation of several CPA3 + granules in MC, freely located in the cytoplasm (indicated by an arrow). (**c**) Melanoma of the skin. Filling the cytoplasm with MC CPA3 + granules. (**d**) Melanoma of the skin. Perinuclear localization of CPA3^+^ granules of varying degrees of maturity, the formation of type III granules (indicated by an arrow). (**e**–**h**) Melanoma of the skin. Different variants of the accumulation of mature CPA3^+^ granules in the cytoplasm of MC. (**i**–**k**) Melanoma of the skin. Denucleation of MC with the formation of a nuclear-free CPA3-positive fragment of the cytoplasm (**k**). (**l**) Skin. Predominant peripheral localization of secretory granules in the cytoplasm of the MC. (**m**) Skin. An elongated mast cell filled with granules along the periphery of the cytoplasm. (**n**) Skin. Peripheral localization of CPA3^+^ granules in the transversely cut elongated part of the mast cell. (**o**) Melanoma of the skin. General view of a mast cell filled with mature CPA3-positive granules of approximately equal size. The protease is located at the periphery of the granule (indicated by the arrow). A granule fusion process occurs, leading to an increase in the size of the larger granule (double arrow) (**p**) Melanoma of the skin. CPA3^+^ MC granules of various sizes, the protease is located at the periphery of the granule (indicated by the arrow). There are areas of cytoplasm free of granules (double arrow). Scale bar: 1 μm (**p’**), 5 μm (the rest).

**Figure 3:**
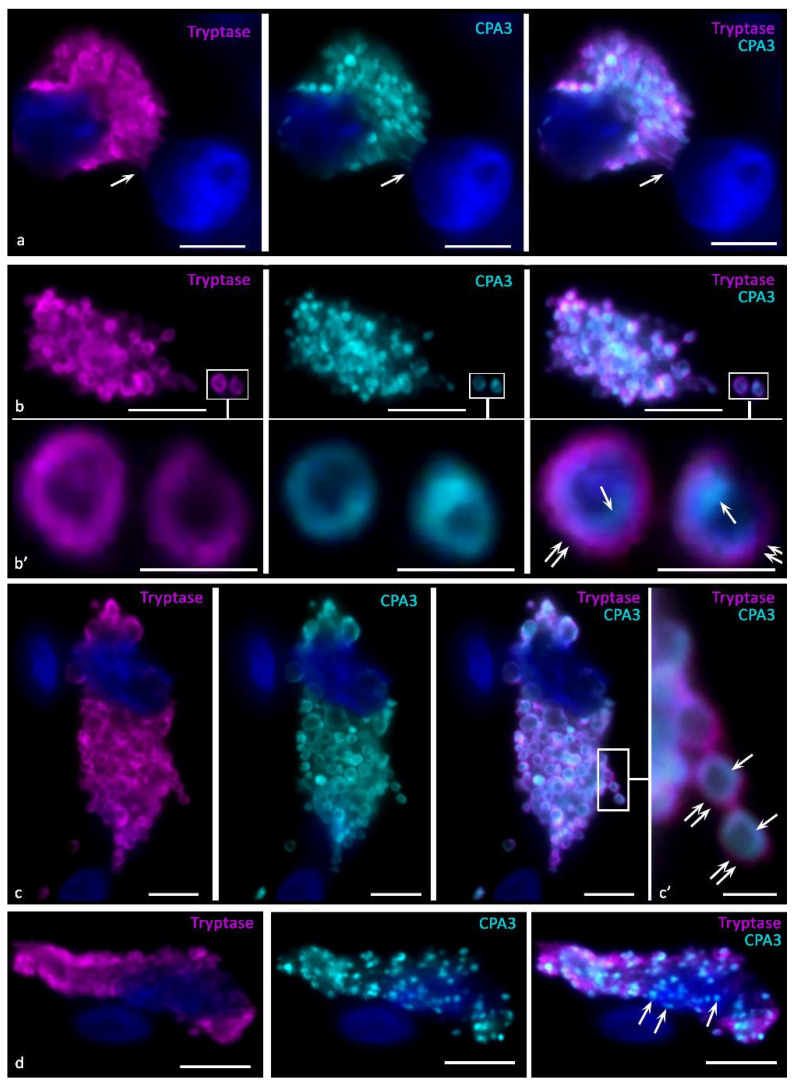
Intragranular localization of CPA3 and tryptase in mast cells of human skin in melanoma. Primary antibodies used: Rabbit polyclonal to CPA3 antibody (ab251696) (AbCam, Cambridge, UK), mouse monoclonal [AA1] to Mast Cell Tryptase antibody (ab2378) (AbCam). Secondary antibodies used: Goat anti-rabbit IgG Ab (#A-11034), Invitrogen, Darmstadt, Germany, label-Cy3; Goat anti-mouse IgG Ab (#A21236), Invitrogen, label-Alexa Fluor 647. (**a**) Tumor microenvironment. Colocalization of tryptase and CPA3 in mast cell granules, some of which are concentrated in the area of contact with the nucleus of a neighboring cell (indicated by an arrow). (**b**,**c**) General view of tumor associated mast cells. Tryptase and CPA3 are colocalized in the same granules, while in mature granules CPA3 is located medially from tryptase (indicated by an arrow), tryptase is located more peripherally (double arrow). (**b’**,**c’**) Enlarged areas of the image from the corresponding areas of photomicrographs, bounded by a frame. (**d**) CPA3^+^ mast cell secretory granules without tryptase (indicated by an arrow). Scale bar: 1 μm (**b’**,**c’**), 5 μm (the rest).

The functioning of the endosomal recirculating compartment can be considered as an additional tool for a qualitative change in the composition of secreted MC products adequate to the state of the extracellular matrix [83] (Figure 1). Granules of various degrees of maturity can become a source of the formation of protease-containing exosomes, while the inclusion of CPA3 in them is not excluded (Figure 1) [92]. The predominant localization of CPA3 along the periphery of the granules is seen more clearly with fluorescence microscopy, especially when they reach large sizes (Figure 2 and Figure 3). At the same time, a definite regularity should be noted that the localization of CPA3 is more central to tryptase and chymase (Figure 3).

According to the degree of maturity, secretory granules can be subdivided into three types [62,65,83]. Endosomes or lysosomes with luminal vesicles belong to the type I. Electron microscopic examination shows a low degree of condensation of components in type I secretory granules and a predominant content of microvesicles [93]. The Rab5 protein is integrated into the fusion mechanisms of Golgi complex budded progranules with early endosomes, which leads to the formation of a larger hybrid organelle [94] (Figure 1). Further maturation of granules is associated with Rab5-mediated fusion with other secretory granules of varying degrees of maturity, which is accompanied by the selective accumulation of some preformed secretome components. This cycle can be repeated several times, leading to the formation of type II granules with larger sizes and a higher degree of heterotypy in the composition of secretory mediators [93,94]. The difference between the ultrastructure of type II secretory granules from type I is in the formation of an electron-dense nucleus around which multivesicular bodies accumulate [65,93]. The processes of Rab5-mediated homotypic and heterotypic fusion of secretory granules involve the vesicle-associated membrane protein VAMP8 [95].

The ripening process of granules when ripening to type III can be either fast enough or take a longer time [61]. In this case, type II granules are gradually saturated with proteoglycans and preformed components of the secretome, with a characteristic intragranular packing. The formed granules of type III can reach a size of 1 μm and contain secretory products in different proportions to each other. Moreover, mature granules can significantly differ from each other in the arsenal of accumulated mediators and create an individual secretory phenotype of the mast cell. Attention is drawn to the fact that it is possible to change the composition of type III granules by including/removing the necessary components of the secretome. These modifications involve the synaptagmin III protein, which is capable of transporting selective components between maturing secretory granules and the endosomal recirculating compartment [96,97].

Mature granules of MCs can continue to accumulate CPA3 and other specific and nonspecific MC proteases, proteoglycans with other mediators in close relationship (Figure 1). In particular, delivery of histamine and serotonin to granules occurs from the cytosol using the vesicular monoamine transport system. An increase in the content of histamine in granules correlates with an increase in the concentration of specific proteases of endogenous origin, which suggests different mechanisms of its participation in their biogenesis [69,98].

A number of components of secretory granules can come from the extracellular matrix. It is known that the inclusion of tumor necrosis factor, the main protein of eosinophil granules and histamine into granules is possible by the mechanism of endocytosis from cellular and extracellular components of a specific tissue microenvironment [99,100,101].

Localization of carboxypeptidase A3 in granules affects their homeostasis. Experiments on mice with a knocked-out CPA3 gene showed a change in the histochemical properties of mast cells, a decrease in the effect of metachromasia in the absence of ultramicroscopic changes, as well as a close relationship of the CPA3 content with another specific protease—mouse mast cell protease-5 (mMCP-5)—which is an analogue of human chymase [102,103]. At the same time, an increase in the content of CPA3 in MCs was accompanied by an increase in the level of mMCP-5, whereas the lack of mMCP-5 led to the inability to accumulate in CPA3 granules [76]. Taking into account the fact that in human MCs, chymase is usually coordinately expressed at a 1: 1 molar ratio with CPA3, an assumption was made about the possibility of strong interactions between MC-CPA3 and chymase [7,46].

## 4. Cytotopographic Features

**Figure 4:**
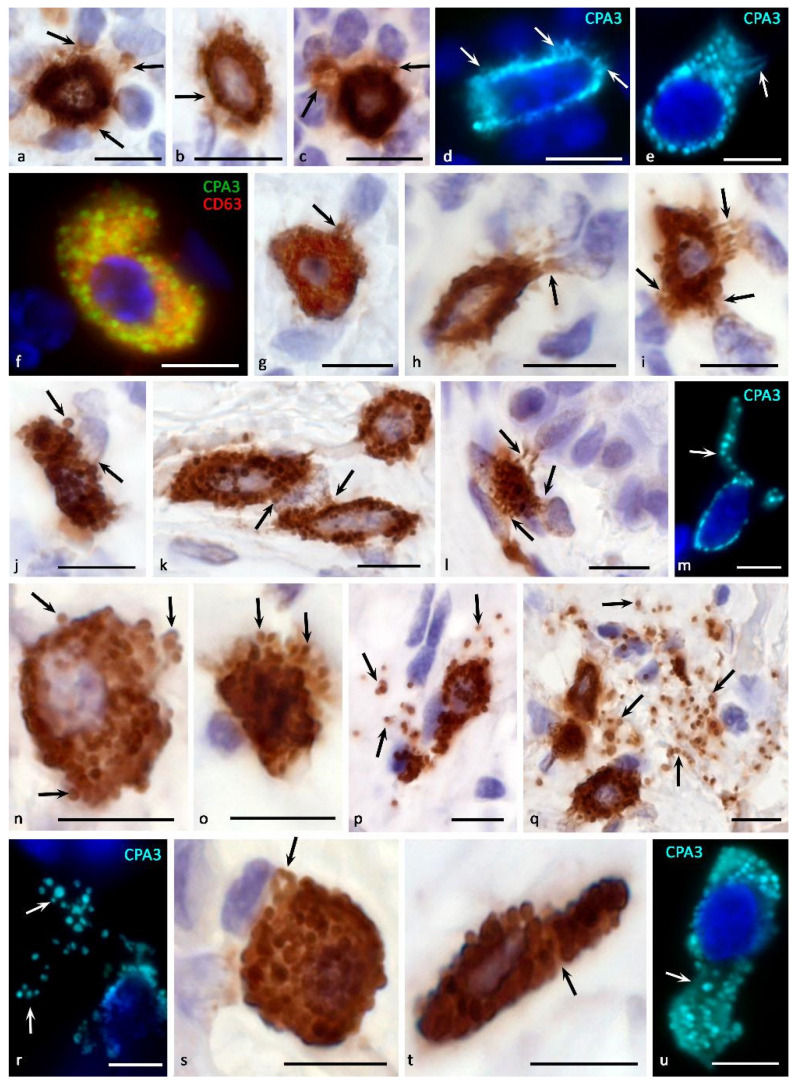
Morphological equivalents of mast cell CPA3 secretory pathways. Primary antibodies used: Rabbit polyclonal to CPA3 antibody (ab251696) (**a**–**u**) (AbCam), and Mouse monoclonal [NK1/C3] to CD63 antibody (ab1318) (**f**) (AbCam). Secondary antibodies used: (**a**–**c**,**g**–**l**,**n**–**q**,**s**,**t**) AmpliStain anti-Rabbit 1-Step HRP (#AS-R1-HRP), SDT GmbH, Baesweiler, Germany, label-HRP. (**d**–**f**,**m**,**r**,**u**) Goat anti-rabbit IgG Ab (#A-11034) Invitrogen, label-Cy3. (**f**) Goat anti-mouse IgG Ab (#A-11029), Invitrogen, label Alexa Fluor 488. (**a**,**b**) Tonsil. Compound CPA3 exocytosis with probable involvement of piecemeal degranulation. Selective accumulation of CPA3 in targets of a specific tissue microenvironment (indicated by an arrow) (**a**), accumulation of protease in the pericellular space of the extracellular matrix (indicated by an arrow) (**b**). (**c**) Tonsil. Formation of MC loci with the most intense CPA3 secretion (indicated by an arrow). (**d**,**e**) Skin. Secretion of CPA3 into the extracellular matrix by the mechanism of compound exocytosis and piecemeal degranulation, with the accumulation of protease in the pericellular space of neighboring cells (indicated by an arrow) (**d**). Formation of a separate MC cytoplasmic locus for CPA3 secretion (indicated by an arrow) (**e**). (**f**) Melanoma of the skin. High content of exosomes in CPA3^+^ mast cell. (**g**,**r**) Morphological variants of targeted degranulation of CPA3 by the mechanism of exocytosis to cellular targets in the specific tissue microenvironment of the tonsilla (**g**–**j**) and skin (**k**–**m**) (indicated by an arrow). (**n**–**p**) Tonsil. The initial stages of mast cell degranulation by the mechanism of exocytosis (**n**,**o**) (indicated by an arrow), leading to the distribution of CPA3 + secretory granules in the extracellular matrix (**p**) (indicated by an arrow). (**q**–**r**) Skin. Active exocytosis of mature secretory MC granules into the extracellular matrix with the formation of loci of the tissue microenvironment with a high content of CPA3^+^ granules (indicated by an arrow). (**s**–**u**) skin process of formation of CPA3^+^ macrovesicles of small (**s**) and large sizes (**t**,**u**) (indicated by an arrow). Scale bar: 5 μm.

**Figure 5:**
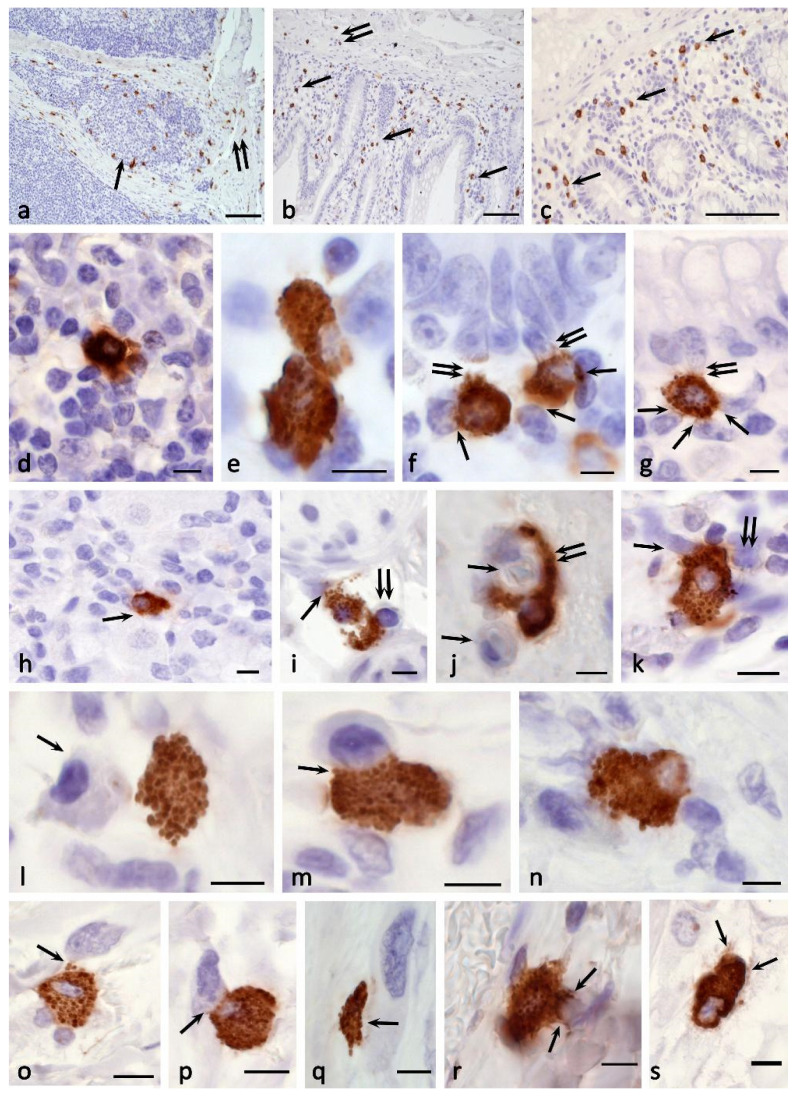
Histotopographic features of the distribution of CPA3 + mast cells in organs. Primary antibodies used: Rabbit polyclonal to CPA3 antibody (ab251696) (AbCam). Secondary antibodies used: AmpliStain anti-Rabbit 1-Step HRP (#AS-R1-HRP), SDT GmbH, label-HRP. (**a**) Tonsil. Localization of CPA3^+^ MC in the lymphoid tissue (indicated by the arrow) and stroma (double arrow) of the organ. (**b**,**c**) The jejunum. The location of CPA3^+^ MC in the mucosa (indicated by the arrow) and submucosa (indicated by the double arrow). (**d**) Tonsilla. CPA3^+^ mast cell in interfollicular lymphoid tissue. (**e**–**g**) Jejunum. CPA3^+^ MC contacting in the lamina propria of the mucous (**e**), interaction with stromal cells (indicated by an arrow) and epithelium (indicated by a double arrow) of the mucous (**f**,**g**). (**h**–**i**) Stomach. CPA3^+^ MC in the stroma of the lamina propria of mucosa, colocalization with the parietal cell of the fundic gland (arrow) and stromal cells (**h**). CPA3^+^ MC degranulates in the direction of the perineurium (indicated by the arrow) and the lymphocyte (double arrow) of the submucosa (**i**). (**j**,**k**) Skin. Histotopographically, CPA3^+^ MC is colocalized with two capillaries (indicated by an arrow); however, CPA3 + secretory material selectively surrounds only one of them over a large area of the basement membrane of the endothelium (indicated by a double arrow) (**j**). MC is colocalized with stromal cells; it is obvious that CPA3 can affect several cells simultaneously, including fibroblast (indicated by an arrow) and an immunocompetent cell (**k**). (**l**) Small intestine. The location of CPA3^+^ MC near the macrophage (indicated by the arrow). (**m**) Small intestine. Interaction of CPA3^+^ MC and an activated lymphocyte (indicated by an arrow). (**n**) Skin. CPA3 + MC in the tumor microenvironment of melanoma contacts with immunocompetent cells and fibroblast (indicated by an arrow). (**o**–**q**) Melanoma of the skin. Various variants of colocalization of CPA3^+^ MC with fibroblasts of the stromal component (indicated by an arrow). Directed exocytosis of CPA3^+^ secretory granules (**o**), as well as morphological evidence of transgranulation, «kiss-and-run» degranulation and formation of exosomes (possibly) for the secretion of CPA3 to biological targets (**p**–**q**) are determined. (**r**–**s**) Skin melanoma. Active participation of CPA3 + MC in remodeling of the fibrous (**r**) and amorphous (**s**) components of the extracellular matrix of the tumor microenvironment (indicated by the arrow). Scale bar: 100 μm (**a**–**c**), 5 μm (the rest).

The content of CPA3 in granules and the number of CPA3^+^ granules in the cytoplasm of MC show a wide variability, from absence or low content to full filling of the cell volume (Figure 2, Figure 3, Figure 4 and Figure 5). Localization of carboxypeptidases in MC granules is convincingly demonstrated by immunohistochemical protocols and can be used to characterize the structure of the MC population (Figure 2, Figure 3, Figure 4 and Figure 5) [84]. The technological features of immunomorphological staining make it possible to assess the cellular and intragranular localization of CPA3 in MCs by other specific proteases (Figure 2), as well as co-localization of CPA3-positive MCs with components of a specific tissue microenvironment.

The ultrastructural compartmentalization of CPA3 in granules is an object for discussion and study. According to our assumptions, the enzyme forms macromolecular complexes with glycosaminoglycans in granules in close relationship with tryptase and chymase. At the same time, selective interaction of CPA3 with certain proteoglycans of granules is possible. In particular, this judgment was made in the analysis of secretory granules after degranulation of MC, in which chymase with CPA3, or only tryptase was detected [38,39,43]. At the same time, we have shown that tryptase is also detected in CPA3^+^ granules after degranulation [84] (Figure 3b,c). Thus, the phenotype of specific proteases of secreted granules can be significantly variable depending on the state of the specific microenvironment. This is confirmed in works on the immunomorphological study of MCs, which showed the presence of granules with different qualitative composition [84] and the need for co-localization of CPA3 with chymase for its deposition [7,32].

Regardless of the presence of tryptase or chymase, CPA3 can be contained in mast cell granules in high amounts. Depending on the level of biogenesis of the granules, CPA3 can occupy the central region of the granules, or be in the form of a ring around the periphery (Figure 1, Figure 2 and Figure 3). In any case, it should be noted that co-localization of MC-CPA3 in granules with other specific proteases occurs in the inner side of the granule, sometimes occupying the entire area with small granules (Figure 3).

The localization of specific proteases in MC granules was also assessed by electronic immunohistochemistry [104]. The MCs of the mucosal subpopulation (with tryptase expression) had granules of the “roll-like ultrastructural phenotype”. In this case, the beads contained scrolls-like parallel concentric plates, in some cases surrounding an electron-dense core [104]. In the connective tissue subpopulation of MCs with simultaneous expression of tryptase and chymase, granules had a specific ultrastructural phenotype, “scrolls poor”, which is characterized by an amorphous central region surrounded on the periphery by parallel plate-like structures [105]. The central zone of such granules could contain an electron-dense material in the form of a lattice. At the same time, granules of immature MCs with simultaneous expression of tryptase and chymase contained one or more amorphous electron-dense nuclei [104].

In other studies, one can find results indicating a high degree of polymorphism of electron-contrast figures in granules in the form of scrolls, crystals, filamentous formations, beads, and their combinations [106,107]. Obviously, these structures may be due to the mutual arrangement of CPA3 with tryptase and chymase, as well as other preformed secretome components. Previous studies have shown the fundamental possibility of CPA3 co-localization with chymase, tryptase, and cathepsin G [105]. In mice with a knockout of the CPA3 gene, granule defects were not detected at the ultrastructural level, however, an increased solubility of proteoglycans of MC granules was observed [102]. It is possible that the absence of CPA3 led to a change in the spatial configuration of serglycin with specific MC proteases, and, as a result, a change in the histochemical staining of the granules.

Our studies using multiplex immunohistochemistry technologies [84] showed that proteases can be located both in different granules and in the same ones, occupying similar loci in the intragranular matrix (Figure 3). The ratio of MCs with immunophenotypes CPA3+Tr+Ch+, CPA3+Tr+Ch− or CPA3+Tr−Ch+ in an organ-specific population and the assessment of the activity of CPA3 secretion is important for determining the state of the tissue microenvironment, having the diagnostic significance of both adaptive and pathological changes [3,84]. In addition, at the level of a single MC, one should take into account the localization of carboxypeptidase-positive granules, which can be located perinuclearly (Figure 2a–d), fill the entire volume of the cytoplasm with varying degrees of density (Figure 2e–k) or occupy a peripheral position, being localized in the periplasmalemma region (Figure 2l,m). At the same time, if a mast cell has an elongated cytoplasm, its nuclear-free part may resemble a process, in which the peripheral localization of granules is also preserved (Figure 2n). This pattern was also found in MC with mastocytosis [84]. Further study of the molecular morphology of intragranular co-localization of CPA3 with other secretome components can most likely lead to the verification of new diagnostic markers, including socially significant diseases.

It can be assumed that CPA3 is required for the biogenesis of both tryptase and chymase in MCs. At the same time, there is no certainty that immunohistochemical staining takes into account the entire volume of the mast cell population in the organ. In particular, in experiments on rats using a combined histochemical protocol, we have shown that a large number of mast cells in the skin can be detected on the basis of metachromasia. This suggests that the total pool of MCs in the organ may escape the attention of researchers if they rely only on the content of one of the specific proteases [91].

A feature that distinguishes CPA3 from enzymatic analogues of carboxypeptidases in the pancreas is the high content of positively charged amino acid residues. This may mediate CPA3 interactions with the negatively charged serglycin proteoglycans in complex with heparin or chondroitin sulfate, which are present in high amounts in MC secretory granules [108]. A high affinity of CPA3 binding to heparin was shown, while there is evidence of a difference between heparin complexes with CPA3 and chymase from heparin-tryptase complexes [104,109]. In addition, it is possible that after degranulation, chymase and CPA3 can be retained on the surface of the MC for some time [110].

Clear evidence of the need for heparin in regulating the accumulation and storage of CPA3 was obtained after experiments on the inactivation of the gene of a key enzyme in the biosynthesis of heparin-N-deacetylase/N-sulfotransferase-2 [111,112]. In these animals, the CPA3 protein was almost completely absent, despite the maintenance of a high level of the corresponding mRNA. The importance of heparin and serglycin for CPA3 storage has been shown [72,78,112].

## 5. Mechanisms of Secretion

It can be assumed that the storage of CPA3 in an active form, ready for the realization of physiological effects, allows MC to almost instantly use its enzymatic potential in various secretion methods, including pacemaker degranulation, the kiss-and-run mechanism, mixed exocytosis, etc. (Figure 1 and Figure 4). When CPA3 is released from intragranular storage loci into the extracellular matrix, the preservation of its biological activity may depend on the bonds with heparin, which protects against the action of endogenous inhibitors. Obviously, the methods of secretion of CPA3 into the extracellular matrix determine the features of its biological effects on the structures and cells of the tissue microenvironment. If it is necessary to maintain certain concentrations of CPA3 in the extracellular matrix, its certain level can be created by the background secretion of the protease with the help of gradual (pacemaker) degranulation of MC (Figure 1). The intensity of the “shuttle secretion” is determined by the degree of involvement of MC in the formation of homeostasis of the integrative-metabolic environment. Separation from the parent granule of a microvesicle 30 to 150 nm in size with proteases, proteoglycans, and other mediators occurs as needed and ends with targeted intracellular transport to the plasmalemma with further secretion into the extracellular matrix [113]. In these molecular mechanisms, proteins of the SNAREs group are of central importance, providing the specificity of vesicular transport and fusion of secretory granules with the plasma membrane or other membrane organelles [114]. At the same time, the intensity of the background secretion of CPA3 in MC can be changed in a certain range, depending on the specific state of the local tissue microenvironment of the organ [63,83,115]. This secretory mechanism is most suitable for the entry of a small amount of CPA3 into the extracellular matrix, creating at the right time the required level of protease in a certain tissue locus (Figure 1 and Figure 4a–d). Gradual degranulation may be involved in the cooperation of MCs with each other, coordinating the level of their secretory activity in normal or pathological conditions, in particular, chronic inflammation, allergies, urticaria, Crohn’s disease, and oncogenesis [63].

Secretion of CPA3 by pacemaker degranulation may be the predominant mechanism in MCs. At the same time, the absence of CPA3 in the granules can cause an erroneous idea about the level of expression of this protease in the mast cell. A similar opinion is expressed about CPA3 biogenesis in lung MCs, in which CPA3 secretion supposedly occurred without storage in granules [10]. Secretory pathways of preformed MC secretome components using non-IgE-mediated mechanisms are widely used by MCs in response to infectious agents, toxins, hormones, alarm signals, changes in the integrative-buffer metabolic environment of the tissue microenvironment, and other factors [116].

The “kiss-and-run” secretion mechanism seems to be analogous to gradual degranulation to a certain extent [83] (Figure 1 and Figure 5l,m). In this case, the MC granule approaches the plasma membrane, comes into contact with the plasma membrane, and at the moment of formation of a temporary pore that communicates with the extracellular environment, the protease can be secreted in a volume adequate to the needs of the specific tissue microenvironment at a given time. It is quite possible that the scale of CPA3 secretion of MC with this method of degranulation is more dynamic, increasing the content of protease in the extracellular matrix by more significant values in a shorter time. In this mechanism, calcium is important, the level of which is a tool for influencing the secretory activity of MC.

There is morphological evidence of the possibility of CPA3 influence on the elements of the tissue microenvironment through the mechanism of transgranulation. This molecular algorithm is implemented at the closest approach of MC to other cells, during which microprotrusions are formed on a certain area of the plasma membrane, closely adjacent to the plasmalemma of another cell. Transgranulation between MCs and fibroblasts, capillary endothelium, and neurons has been described [63]. It is possible to interpret the inductive effect of CPA3 when MC is attached to other cells and extracellular structures of a specific tissue microenvironment, including immunocompetent cells, epithelia, fibroblasts, collagen fibers, etc. [117] (Figure 5).

In MCs, degranulation of specific proteases is possible through the formation of extracellular vesicles, incl. exosomes [63,92,118] (Figure 1). Exosomes can contain various types of RNA, including mRNA and microRNA, which are transferred to CD34+ undifferentiated cells and immature MCs, dendritic cells, T- and B-lymphocytes, having a corresponding effect on the processes of development, migration and biosynthesis of various mediators [118]. The possibility of CPA3 liberalization from MC within exosomes significantly expands the regulatory potential of the protease in relation to the cellular and non-cellular components of a specific tissue microenvironment [92]. Due to the known complexes of chymase with CPA3, secretion of the latter with the help of exosomes is quite possible. The varying degree of involvement of the exosomal mechanism in the CPA3 secretory pathways is confirmed by the results of multiplex immunohistochemical staining (Figure 4f).

To increase the volume and, in some cases, the rate of entry of MC CPA3 into the extracellular matrix, the mechanisms of secretion of individual granules or their combination as part of a budding fragment of the MC cytoplasm-“macrovesicles” can be used (Figure 4g–u) [48,73,119]. Both variants of degranulation differ from those described above, first of all, by the certain autonomy of the secreted formations. Possessing a significant resource of specific proteases, secretory granules are able to remain in the extracellular matrix for a long time after being removed from the MC, exerting a selective effect on a number of cells and extracellular structures. In some cases, the directed secretion of CPA3 towards the nuclear structures of other cells becomes apparent (Figure 4g–l). The study of CPA3 in this aspect seems to be of particular interest after the discovery of the ability of tryptase to have regulatory effects on nuclear histones and thereby influence the proliferative activity of cells [120,121,122]. Moreover, there is clear immunohistochemical evidence of tryptase and CPA3 co-localization in the case of directed secretion of proteases to the nuclei of other cells (Figure 3a). However, the significance of CPA3 in this phenomenon remains to be elucidated.

Sometimes CPA3^+^ secretory granules literally infiltrate certain loci of the tissue microenvironment of the organ due to active MC degranulation (Figure 4n–r). On the one hand, this method of secretion allows not only to create significant amounts of CPA3 in MC granules, but also to form an autonomous substance with certain biological properties. Thus, the granules individually or as part of larger cytoplasmic fragments of the MC can represent independent postcellular structures with their own regulatory potential [59,123]. In addition, ultramicroscopic evidence of the presence of RNA and ribosomes in the composition of secretory granules allows the continuation of biosynthetic activity after leaving the MC cytoplasm [82,124].

The possibility of the protease transferring as a part of postcellular structures to targets allows specific biological effects to be carried out at considerable distances from the site of initial degranulation, and, in some cases, to create quite extensive inductive fields with a high concentration of MC CPA3, in particular, in patients with melanoma in the skin or in the tonsil with chronic inflammation [9] (Figure 4p–r). Compared to individual granules, fragments of the MC cytoplasm can be considered as a prolongation of the effector action of specific proteases [48,73,119]. In addition, the possibility of active migration to such formations of other connective tissue cells, for example, leukocytes, for endocytosis of the necessary components or obtaining the regulatory effects of the protease deserves attention.

In pathology, other pathways of MC secretory activity can also be realized. Among the well-studied is anaphylactic degranulation of MC, accompanied by massive excretion of granules into the extracellular matrix (Figure 1) [83]. Binding of immunoglobulin E to FcεRI receptors results in tyrosine phosphorylation and activation of signaling proteins. The rapid transport of granules to the plasmalemma ends with fusion with it with the participation of the SNARE complex (including proteins SNAP-23, VAMP-7, VAMP-8, syntaxin-4, etc.) with massive extrusion of the contents into the extracellular matrix (Figure 1) [83,125]. The rapid activation process primarily affects the granules adjacent to the plasmalemma; only after some time the rest of granules are involved in the reaction. Rounded protrusions up to 1.5 μm in size can form on the cell surface, the number of which correlates with degranulation [126]. Intense fusion of granules may be accompanied by the formation of intracellular highways for the excretion of secretome products in the form of canal-like passages ending in pores (Figure 3). As a result, a large amount of chymase and also other proteases and mediators enter the extracellular matrix with the risk of generalization of the process.

## 6. Biological Effects

Despite the abundance of CPA3 in MCs, there is currently a lack of knowledge about its biological effects compared to other specific mast cell proteases, tryptase and chymase. The involvement of CPA3 in innate immunity is best known. In particular, the involvement of the protease in the body’s defense against snake venom and some toxins has been shown [14,127,128,129]. Inhibitors of CPA3 MC produced by the nematode Ascaris significantly increase the survival of the parasite during infection. MCs are key players in protection against the vasoconstrictor peptide endothelin 1 (ET-1), which plays an important pathogenetic role in sepsis, skin itch, fibrosis, etc. [14,130,131,132,133]. In addition, by limiting the biological effects of ET-1 with the help of CPA3, MCs have important effects on the state of the lung parenchyma and systemic blood flow [131,134].

It has also been shown that CPA3 in MC indirectly has vasodilatory and bronchodilatory effects due to the ability to transform C4 leukotriene into F4 leukotriene and thereby reduce the likelihood of the formation of D4 and E4 leukotrienes with more powerful broncho- and vasoconstrictive effects [135].

It is known that CPA3 can cleave neurotensin, kinetensin, neuromedin N and angiotensin I [12,13,15,127,136]. Degradation of sarafotoxin, neurotensin, and ET-1 leads to the loss of their biological activity, while degradation of apolipoprotein B can promote the fusion of low-density lipoproteins and, thus, the formation of atherosclerotic plaques [137,138,139]. The lack of CPA3 in blood plasma has been noted as a risk factor for the development of diseases of the cardiovascular system, including elements of the microvasculature [24].

The functional significance of the CPA3 complex with chymase has been considered under aspects of more efficient degradation of the substrate by the co-localization of proteases [32,130,139]. In addition, chymase and CPA3 can also interact in the enzymatic cleavage of angiotensin II [13], in which each of the enzymes has its own catalytic activity with respect to specific substrate proteins.

A close morphological co-localization of chymase and CPA3 can persist after MC degranulation and take part in the conveyor cleavage of proteins or other peptide targets of the extracellular matrix, requiring the simultaneous presence of endo- and exopeptidases. The importance of CPA3 in the biogenesis of the fibrous component of the extracellular matrix and the remodeling of the extracellular matrix is assumed. On the one hand, the CPA3-chymase complex can induce an increase in the mitotic activity of fibroblasts along with their biosynthetic potential. On the other hand, this complex or proteases individually can take part in the modification of procollagen molecules, inducing the formation of collagen fibrils [5,140]. The conducted studies indicate the active participation of MCs in the mechanisms of fibrillogenesis, which is manifested by an inductive effect on the formation of the fibrous component of the tissue microenvironment, primarily next to the cellular representatives of fibroblastic differon [117] (Figure 5o–q). The presence of reticular fibers or points of initiation of fibrillogenesis was also shown in close proximity to the plasmalemma of the mammary gland [117]. These aspects are indirectly confirmed by studies on the modeling of adhesive processes in the abdominal cavity on laboratory animals, as well as in the study of lungs subject to chronic inflammation or fibrosis and in kidneys [10,15,141].

Tryptase has recently been shown to have epigenetic effects due to its influence on the state of nuclear histones and DNA stabilization [120,121,122]. The close co-localization of CPA3 with tryptase leads to some interest in the extent to which exopeptidase is involved in these effects (Figure 3a).

In mast cell-deficient Cpa3Cre/+ mice, defective bone repair was found, due to a decrease in the amount of MC, a delay in revascularization, accumulation and mineralization of the intercellular substance of the newly formed bone tissue, a change in the activity of osteoclasts and macrophages [20]. The detection of changes in the expression of CPA3 in many diseases highlights the existence of many other points of application of the protease, the study of which in the future will help identify new molecular targets for targeted therapy to improve the effectiveness of treatment [23,26,27,142,143,144].

## 7. Pancreatic Carboxypeptidases in Mast Cells: Myth or Reality?

**Figure 6:**
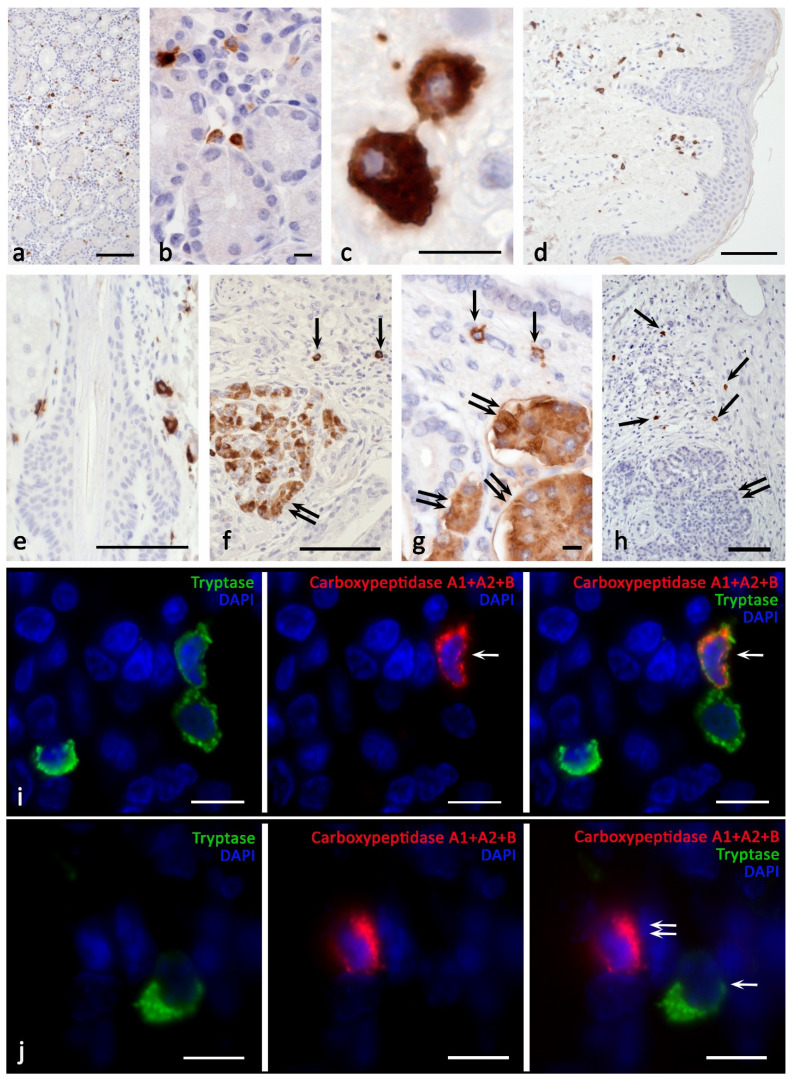
Carboxypeptidases in human mast cells. Primary antibodies used: (**a**–**g**,**i**,**j**) Rabbit monoclonal [EPR12087(B)] to Carboxypeptidase A1+A2+B (ab181146) (AbCam). (**h**) Rabbit polyclonal to CPA3 antibody (ab251696) (AbCam). Secondary antibodies used: AmpliStain anti-Rabbit 1-Step HRP (#AS-R1-HRP), SDT GmbH, label-HRP. (**a**–**c**) Stomach. There is a positive reaction of mast cells of the mucous to the carboxypeptidases of the pancreas. (**d**,**e**) Skin. There is a staining of pancreatic carboxypeptidases in mast cells in the dermis (**d**) and hair follicle (**e**). (**f**–**g**) Pancreas. Pancreatic carboxypeptidases are detected both in mast cells (indicated by an arrow) and pancreatic exocrinocytes (indicated by a double arrow). (**h**) Pancreas. CPA3 is contained in mast cells (indicated by an arrow) and is absent in pancreatic exocrinocytes (double arrow). (**i**) Stomach. A tryptase-positive MC containing pancreatic carboxypeptidases (indicated by an arrow) is detected. (**j**) Stomach. A cell positive for pancreatic carboxypeptidase is visualized (arrow) and tryptase-positive mast cell is visible (double arrow). Scale bar: 100 μm (**a**,**d**,**e**,**f**,**h**), 10 μm (the rest).

In frames of the study of this issue, we drew attention to a large amount of molecular genetic data indicating the isolation in the process of evolution of the CPA3 gene from other carboxypeptidases, including the pancreas. CPA3 belongs to the M14A metallocarboxypeptidase subfamily, which, among others, also includes the digestive enzymes CPA1, CPA2, CPB1, and CPO [11]. There are no indiations in the literature on the detection of pancreatic carboxypeptidases in MC, but there are also no morphological evidences of their absence. We first tried to detect CPA1 in MC (using the primary Anti-Carboxypeptidase A antibody [EPR12086] #ab173283) by staining bone marrow, skin, liver, prostate, and bladder, but the results were negative. However, the use of the Anti-Carboxypeptidase A1+A2+B antibody [EPR12087(B)] #ab181146 led to a convincing detection of MC not only in the bone marrow of healthy people and patients with mastocytosis [84], but also in the stomach (Figure 6a–c), skin (Figure 6d,e), liver, prostate, bladder, and pancreas. It is important to note that pancreatic exocrinocytes were also successfully stained, which methodically confirms the adequacy of the antibodies used and the presence of carboxypeptidases B1 or A2 in mast cells (Figure 6f,g). We also verified that CPA3 antibodies did not label the exocrine pancreas (Figure 6h). Double immunolabeling showed co-localization of pancreatic carboxypeptidases and tryptase only in a portion of the MC population with organ-specific features (Figure 6i). In addition, cells containing pancreatic carboxypeptidases without tryptase or chymase expression were detected (Figure 6j). Intracellular localization of pancreatic carboxypeptidases had some differences from CPA3 cytotopography, in particular, they were more often detected in the intergranular cytoplasm of MCs. In the case of intragranular localization, they could occupy the entire central region of the granule (Figure 6c,i). At the same time, the identified feature requires separate consideration.

Detection of carboxypeptidases A2 or B in MCs can be explained by a common precursor (ancestor) of CPB and CPA3 in MC, diverged by gene duplication from the lineage leading to CPA, and then underwent another gene duplication to form separate but similar gene structures for CPA3 and CPB during time of early tetrapod evolution [11]. That is why, despite the presence of a CPA-like substrate-binding pocket and specific enzymatic activity, CPA3 is more similar to CPB in protein structure and molecular genetic features [7,32,145,146]. CPA is unique among carboxypeptidases in having a CPA-like substrate-binding pocket and enzymatic activity despite overall protein and gene structures more similar to CPB [37]. The possibility of the origin of CPA3 in MCs from a common precursor with pancreatic carboxypeptidases has been shown in many studies. Indeed, with the exception of intron 3, the localization of the remaining introns in the CPA3 gene is highly conserved, which indicates an origin from a common precursor gene with other carboxypeptidases (CPA1, CPA2, and CPB). In terms of exon-intron organization, human CPA3 is more similar to pancreatic CPB compared to CPA1 and CPA2. In turn, a common origin of the pancreatic CPB gene with the CPA1 and CPA2 genes was found [7,32,37,46,147,148]. Phylogenetic analysis has shown that CPA3 in MCs is more closely related to CPB1 and CPO than to the carboxypeptidases CPA1, CPA2, CPA4, CPA5, and CPA6. Co-localization of CPA3 and CPB1 in the same locus and with the same flanking genes was found in all tetrapods and ray-finned fish, for which MCs are characterized by the expression of only CPB1 [11].

As a representative of metallocarboxypeptidases, zinc-dependent MC exoprotease hydrolyzes C-terminal amino acid residues [149]. However, it should be emphasized that the enzymatic activity of pancreatic CPA and CPA3 of MCs differ, in particular, in relation to the hydrolysis of bradykinin and substance P [36]. Mouse MC CPA3 preferentially removes bulky aromatic amino acids, similar to CPA2 [16]. Rat CPA3 has been shown to enzymatically act on typical substrates for pancreatic CPA, indicating its CPA-like activity [38]. This is also consistent with the data on the effects of angiotensin I hydrolysis of CPA3, CPA and CPB1 in MC [36,139]. However, compared to CPB1, carboxypeptidase CPA3 does not cleave at cites after aromatic amino acids; instead it cleaves after the basic amino acids Arg and Lys [11]. The fundamental possibility of detecting pancreatic carboxypeptidases in the granules and cytoplasm of MCs may open up new prospects for the study of the processing of specific MC proteases and lead to the discovery of new targets for directed changes in the secretory phenotype of MCs.

## 8. Conclusions

The discussed features of CPA3 processing, accumulation, and secretory pathways, together with the facts of its abundant content in MCs, allow us to consider this protease an important characteristic of the MC protease phenotype and one of the key components of the organ-specific characteristics of their population. Features of intracellular co-localization of CPA3 with other specific mast cell proteases, as well as the spatial distribution of CPA3^+^ MCls in tissues with the establishment of patterns of their co-localization with immunocompetent and stromal cells form new ideas about the fundamental mechanisms of regulation of the state of the integrative-buffer metabolic environment and the extracellular matrix. At the same time, it is obvious that our knowledge of the biological effects of this protease compared to tryptase and chymase is significantly limited. Future studies will shed light on the mechanisms of involvement of CPA3 in the pathogenesis of various diseases, and, most importantly, the mechanisms of their origin at the level of a specific tissue microenvironment. Thus, CPA3 is a promising target in translational medicine, both in terms of diagnostic value and as a potential target for targeted therapy.

## Data Availability

All data and materials are available upon reasonable request. Address to I.B. (email: buchwalow@pathologie-hh.de) or M.T. (email: mtiemann@hp-hamburg.de) Institute for Hematopathology, Hamburg, Germany.
